# Laboured reading and musculoskeletal pain in school children - the role of lifestyle behaviour and eye wear: a cross-sectional study

**DOI:** 10.1186/s12887-022-03465-1

**Published:** 2022-07-13

**Authors:** Hanne-Mari Schiøtz Thorud, Randi Mork, Cecilie Onshuus Bjørset, Stuart J. Gilson, Lene A. Hagen, Trine Langaas, Hilde R. Pedersen, Ellen Svarverud, Gro Horgen Vikesdal, Rigmor C. Baraas

**Affiliations:** National Centre for Optics, Vision and Eye Care, Department of Optometry, Radiography and Lighting Design, University of South-Eastern Norway, Kongsberg, Norway

**Keywords:** Headache, Adolescent, Sex differences, Eye, Vision, Glasses, Screen, Digital, Physical activity

## Abstract

**Background:**

Lifestyle behaviour in children and adolescents has become increasingly sedentary and occupied with digital work. Concurrently, there has been an increase in the prevalence of headache, neck- and low back pain, which are leading causes of disability globally. Extensive near work and use of digital devices are demanding for both the visual system and the upper body head-stabilizing musculature. Uncorrected vision problems are present in up to 40% of Nordic school children, and a lack of corrective eye wear may cause eyestrain, musculoskeletal pain and headache. The aim of this study was to investigate associations between laboured reading, musculoskeletal pain, uncorrected vision, and lifestyle behaviours in children and adolescents.

**Methods:**

This was a cross-sectional study with a total of 192 Norwegian school children aged 10–11 and 15–16 years. As a part of a school vision testing program, the children completed an online questionnaire about general and ocular health, socioeconomic status, academic ambition, near work and related symptoms, upper body musculoskeletal pain, and physical and outdoor activities.

**Results:**

The 15–16-year-olds had a more indoor, sedentary, digital-based lifestyle with higher academic demands, compared with the 10–11-year-olds. Concurrently, reading became more laboured and upper body musculoskeletal pain increased with age. Girls reported more symptoms, higher academic ambitions, and more time spent on schoolwork and reading, compared with boys. Non-compliance in wearing prescribed eye wear (glasses or contact lenses), increased use of near digital devices, and experiencing visual stress (glare) were positively associated with laboured reading and upper body musculoskeletal pain.

**Conclusions:**

A screen-based lifestyle with high academic demands challenges the ability to sustain long hours of static, intensive near work. Extensive near work tires the visual system and upper body musculature and provokes laboured reading and musculoskeletal pain symptoms. This study emphasizes the importance of regular eye examinations in school children, and the need to raise awareness among children, parents, and school- and health personnel about the importance of optimal vision and visual environment for academic performance and health.

## Introduction

Globally, children and adolescents have increasingly adopted a more sedentary lifestyle, and they have become more occupied with digital devices in pace with the digital revolution during the last 30–40 years [1]. Additionally, the COVID-19 pandemic has, for many individuals, led to detriments in physical activity and a further increase in sedentary behaviour and use of near digital devices [2, 3]. Sedentary behaviour increases from childhood to adulthood [4]. Concurrently, the prevalence of headache, neck and low back pain increases, and these conditions are leading causes of sickness absence globally [5–8]. Further, in the age group 10–24 years both headache disorders and low back pain have increased during the last 30 years [8]. Near tasks, such as use of near digital devices, require high activity in extra- and intraocular muscles and precise coordination between the visual system and the head-stabilizing muscles. Therefore, uncorrected vision and lack of proper corrective eye wear, will induce eyestrain, upper body muscle tension, and unhealthy, static postures (such as forward bent head). This in turn may cause headache and neck, shoulder, and back pain [9–18]. Among Nordic school children, as many as 40% have uncorrected vision, lacking proper corrective eye wear for satisfactory and effortless vision during tasks at near (reading, writing, screen use) or far (reading a blackboard) [19–22]. Refractive errors, poor accommodation (reduced ability to focus at near), and binocular dysfunctions (poor coordination between the two eyes), have been associated with headache and upper body musculoskeletal pain in children and adolescents [14, 17, 20, 22–25]. Furthermore, studies in children and adolescents indicate that physical inactivity, obesity, sleeping problems, prolonged near work and screen use, socioeconomic and psychosocial factors, and female sex, are risk factors involved in the development of spinal pain and headache [16, 26–43]. Importantly, neck, shoulder and back pain and headache can lead to reduced daily functioning and academic performance, and impair recreational activities, thereby constituting a major health problem [41, 44–47]. Additionally, uncorrected vision and lack of proper corrective eye wear may impact the child’s ability to sustain near tasks over time, such as reading and writing, and thus reducing academic performance [22]. Optimal vision is essential for developing cognitive abilities associated with learning to read and write [48–56]. Wearing glasses for one school year, when these are needed, compared to not wearing them, has been reported to advance the child’s knowledge equivalent of one semester’s learning [57, 58]. Cognition and academic performance have also been shown to increase with physical activity interventions and decrease with low motor performance and recurrent pain problems [59–63].

The purpose of this study was to investigate associations between symptoms of laboured reading, musculoskeletal pain, uncorrected vision, and lifestyle behaviours in children and adolescents. In this study ‘uncorrected vision’ was defined as common vision problems that can be fully corrected with eye wear (glasses or contact lenses), including refractive errors and accommodative and binocular dysfunctions.

## Methods

### Participants

This was a cross-sectional study in 10–11 and 15–16-year-old children (5^th^ and 10^th^ grade) at three schools in Kongsberg municipality, Norway, during the school year 2016–2017. Kongsberg municipality has a population of 1643 per sq. km, and is representative of the Norwegian population regarding public health and socio-demographic status [64, 65]. Sample size was calculated with a test power of 80%, a significance level of 5% (two-tailed) and standard errors based on previous musculoskeletal pain recordings [16]. Musculoskeletal pain was a main variable to investigate in the current study, and the power analysis showed that a total of 184 participants should suffice to identify a 40% difference in musculoskeletal pain between two independent groups. All 200 children attending 5^th^ and 10^th^ grade were invited to participate. There were no other inclusion or exclusion criteria. Written informed consent was obtained from 192 (96%) children and their parents after explanation of the nature and possible consequences of the study. All children in this study participated in the vision testing program offered to school children in 2^nd^, 5^th^ and 10^th^ grade in Kongsberg municipality, Norway. The vision testing program is organized by National Centre for Optics, Vision and Eye Care (NCOVE) at the University of South-Eastern Norway in collaboration with the municipality. The vision testing program consists of history taking and preliminary tests for assessing visual status, including visual acuity and cover test for distance and near, amplitude of accommodation, near point of convergence, motility, colour vision, and stereoacuity. Retinoscopy and autorefraction with and without cycloplegia and biometrical measures of the eye are also performed. Children with an uncorrected vision problem (according to international clinical guidelines [66, 67]) are referred for a full eye examination either at NCOVE or their private optometrist.

Of the 192 children included, 51 children attended 5^th^ grade (10–11-year-olds, 53% girls), and 141 attended 10^th^ grade (15–16-year-olds, 53% girls). In 5^th^ grade, 5 (10%) of the children reported to use daily medication due to; asthma (2 children), vitamin D and iron deficiency (1 child), unknown (2 children). In 10^th^ grade 27 (19%) of the children reported to use daily medication; allergy and asthma (9 children), attention-deficit/hyperactivity disorder (ADD/ADHD) (6 children), vitamin and mineral supplements (6 children), oral contraceptives (4 children), acne vulgaris (4 children), hypothyroidism (1 child), chronic fatigue syndrome (CFS/ME) (1 child). In the whole sample, one (0.5%) child reported congenital cataract, two (1%) reported previous strabismus surgery, and 12 (6%) reported to have used eye patches due to amblyopia or strabismus.

### Questionnaire

The children completed an online questionnaire on their school tablet (iPad, Apple Inc.) in class, during school hours (March 2017), and both a teacher and a member of the project group (HMST, RM, COB, LAH, TL, HRP, ES, GHV, RCB) were present to assist with technical difficulties or clarifying questions. The questionnaire was completed in approximately 15 min, and consisted of nominal, ordinal and ratio questions related to personalia (age, sex), socioeconomic status (number of books at home), academic ambition in 10^th^ grade (preferred length of future education), daily medication, ocular health, use of glasses/contact lenses, symptoms and difficulties during tasks at near (reading, screen use) and far (blackboard), upper body musculoskeletal symptoms, and time spent on different activities on a typical weekday and day off in early March (winter in Norway); paper-based reading (books etc), screen use, outdoor activities, and physical activity. The list of activities was chosen to elicit information about near and far work, physical activity and indoor versus outdoor activities [68].

All questions regarding symptoms were given a score on a 5-point Likert scale: Never (1), Rarely (2), Sometimes (3), Often (4), Always (5). Total scores were computed by combining related ordinal questions: A total reading symptom score, here defined as ‘laboured reading’, was calculated combining the items ‘difficult to see text in book’, ‘tired eyes’ and ‘headache’ when reading, and ‘difficult to remember read text’ (4; max score 20). A total upper body musculoskeletal symptom score was calculated combining all musculoskeletal symptoms (pain in the neck, shoulders, low back, and arm/wrist/hand; 4; max score 20). Questions regarding time spent on different activities on a typical weekday and day off, were given a score on a 4-point scale: Never (1), Less than 1 hour (2), 1–2 hours (3), 3 hours or more (4). Scores on weekdays and days off were combined. Screen time score was calculated combining the items ‘computer use at home and school’, ‘relaxing with tablet’, ‘tablet use at school and when doing homework’, ‘mobile/Gameboy/Nintendo DS surfing and gaming’, and ‘reading books, blogs, newspapers etc. on mobile’ (5; max score 20). The sports score combined the items ‘sports indoor’ and ‘sports outdoor’ (2; max score 8). Total physical activity score was calculated combining the items ‘physical activity outdoor’ (such as walking, playing, hiking), ‘sports indoor’ and ‘sports outdoor’ (3; max score 12). Outdoor score was calculated combining the items ‘physical activity outdoor’ (such as walking, playing, hiking), ‘sports outdoor’, and ‘relaxing outdoor’ (such as spending time in the garden) (3, max score 12).

### Statistics

Raw data were assessed for normality using Q-Q plots and the Shapiro-Wilk test. Differences in means between groups were tested by one-way analysis of variance (ANOVA). Pearson’s correlation coefficient (*r*) was used to investigate associations between continuous variables. Chi-square independence tests were used to evaluate associations between categorical variables. A statistical difference was set at *p* < 0.05 (two-tailed). Statistical analyses were performed in IBM SPSS Statistics (Version 28, US).

## Results

### Symptoms

Table 1 presents symptom scores in 5^th^ and 10^th^ grade. Reading symptom score (F(1,190) = 6.149, *p* = 0.014) and upper body musculoskeletal symptom score (F(1,190) = 5.811, *p* = 0.017) were significantly higher in the older children, and they were significantly correlated (*r* = 0.471, *n* = 192, *p* < 0.001). Girls had significantly higher reading symptom score (F(1,190) = 6.747, *p* = 0.010) and upper body musculoskeletal symptom score (F(1,190) = 4.838, *p* = 0.029) compared with boys. In girls, shoulder pain increased significantly from 5^th^ to 10^th^ grade (2.4 ± 1.1 vs 1.7 ± 0.9 (mean ± SD); F(1,100) = 9.050, *p* = 0.003).

**Table 1:** Symptom and activity scores in 5^th^ and 10^th^ grade

		5^**th**^ grade (10–11 year)		10^**th**^ grade (15–16 year)	
		Girls (***n*** = 27)	Boys (***n*** = 24)	Girls (***n*** = 75)	Boys (***n*** = 66)
Reading symptom score*^#^		8.2 ± 2.7	6.7 ± 1.9	9.2 ± 3.4	8.2 ± 3.1
Upper body musculoskeletal symptom score*^#^		7.7 ± 2.8	6.7 ± 2.1	8.9 ± 3.0	8.0 ± 3.2
**Screen use symptoms (tablet)**	Tired eyes*^#^	2.3 ± 1.0	1.5 ± 0.7	2.4 ± 1.0	1.9 ± 0.9
	Reflections / Glare*^#^	2.6 ± 1.2	2.1 ± 1.1	3.2 ± 0.9	2.9 ± 1.1
**Difficulties with distance vision**^**#**^		2.1 ± 0.9	1.8 ± 1.2	2.0 ± 1.1	1.7 ± 1.0
Reading on paper*^#^		4.2 ± 1.1	4.1 ±1.1	3.9 ± 1.4	3.4 ± 1.1
**Screen time**	Total*	21.2 ± 4.4	22.2 ± 4.7	26.6 ± 5.2	25.7 ± 4.9
	School work (tablet)*^#^	4.6 ± 1.0	4.4 ± 1.1	8.9 ± 1.3	5.4 ± 1.4
Outdoor*		14.4 ± 3.0	15.0 ± 3.3	13.3 ± 3.8	12.1 ± 4.0
**Physical activity**	Total	14.1 ± 3.3	16.0 ± 3.6	15.0 ± 4.3	13.6 ± 4.1
	Sports	8.7 ± 2.6	10.7 ± 2.5	10.1 ± 3.3	9.0 ± 3.1
	Walking / cycling to school* *n* (%)	21 (78)	20 (83)	44 (59)	35 (53)
	Physical active School breaks* *n* (%)	26 (96)	23 (96)	5 (7)	7 (11)

Data are presented as mean ± SD, except for walking/cycling to school and physical activity during school breaks, which are given as frequencies. Difficulties with distance vision: Seeing text on blackboard. Statistically significant difference overall between the age groups* and sex^#^ at *p* < 0.05

Girls were more bothered with tired eyes (F(1,190) = 22.488, *p* < 0.001) and reflections/glare (F(1,190) = 4.209, *p* = 0.042) during screen use (tablet). The older children also reported more reflections/glare (F(1,190) = 17.178, *p* < 0.001). Tired eyes and experiencing reflections/glare during screen use were significantly correlated (*r* = 0.350, *n* = 192, p < 0.001). Further, tired eyes during screen use were significantly correlated with more laboured reading (*r* = 0.547, *n* = 192, *p* < 0.001) and upper body musculoskeletal pain (*r* = 0.314, *n* = 192, *p* < 0.001). Also, children experiencing reflections/glare during screen use often/always (*n* = 60), reported significantly more laboured reading (reading symptom score; F(1,190) = 12.967, *p* < 0.001) and upper body musculoskeletal pain (symptom score; F(1,190) = 9.445, *p* = 0.002), compared with the rest of the sample.

### Vision

**Fig. 1:**
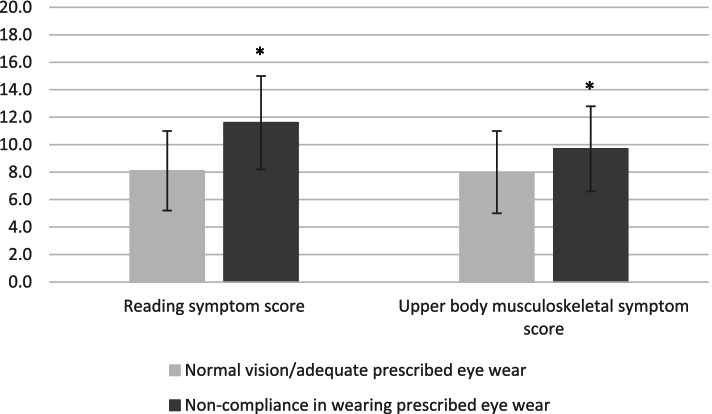
Symptom scores in children with normal vision / adequate prescribed eye wear (*n* = 175) and in children with non-compliance in wearing prescribed eye wear (*n* = 17). Data are presented as mean ± SD. *Statistically significant difference between the groups at *p* < 0.05

The children had previously participated in the vision testing program offered at school (see Methods/Participants). Table 2 describes eye wear use frequencies. The 17 children who did not use their recommended glasses/contact lenses, reported significantly more laboured reading (F(1,190) = 21.457, *p* < 0.001) and upper body musculoskeletal pain (F(1,190) = 4.937, *p* = 0.027) compared with the rest of the sample (Figure 1). In line with this, these children often/always had shoulder pain (χ^2^(1, *n* = 192) = 10.45, *p* = 0.001), arm/wrist/hand pain (χ^2^(1, *n* = 192) = 12.68, *p* < 0.001), tired eyes (χ^2^(1, *n* = 192) = 8.63, *p* = 0.003) and headache (χ^2^(1, *n* = 192) = 17.08, *p* < 0.001) during reading, and tired eyes during screen use (χ^2^(1, *n* = 192) = 5.64, *p* = 0.018), at a significantly higher frequency compared with the rest of the sample. These 17 children also reported significantly more difficulties with distance vision (seeing text on blackboard) (2.7 ± 1.4 vs 1.8 ± 1.0 (mean ± SD); F(1,190) = 11.869, *p* = 0.001). Refractive error and mode of correction were known for 15 of these 17 participants. All had previously been prescribed eyeglasses; 13 were hyperopes (SER ≥ + 0.50 D) of whom two had astigmatism (≥ 0.75 DC) and one had anisometropia (difference between the eyes ≥ 1.00 D). Two were myopes (SER ≤ - 0.50 D) of whom one also had astigmatism. Five of the 17 children who did not wear the recommended eye wear, reported to take daily medication (allergy and asthma (4 children), ADD/ADHD (1 child), prescription vitamin supplements (1 child), and one child reported to have used eye patch due to amblyopia or strabismus. There were no significant differences in socioeconomic status, academic ambition, and time spent on different activities (including schoolwork (on school tablet) and reading on paper) between these 17 children and the rest of the sample.

**Table 2:** Eye wear use in 5th and 10th grade

		5^**th**^ grade (10–11 year)		10^**th**^ grade (15–16 year)	
		Girls (***n*** = 27) *n* (%)	Boys (***n*** = 24) *n* (%)	Girls (***n*** = 75) *n* (%)	Boys (***n*** = 66) *n* (%)
**Glasses / contact lenses**	Total	14 (52)	7 (29)	24 (32)	15 (23)
	All day wear	5 (19)	4 (17)	9 (12)	10 (15)
	Only for distance	2 (7)	1 (4)	4 (5)	2 (3)
	Only for near*	6 (22)	2 (8)	11 (15)	3 (5)
**Non-compliance**^**a**^		1 (4)	1 (4)	11 (15)	4 (6)

Distance: TV, cinema, blackboard. Near: Reading, writing, screen use. ^a^A total of 17 children reported they had been recommended to wear glasses or contact lenses, based on a previous eye examination with an authorized optometrist, but they did not follow the recommendations. *Overall, significantly more girls wore glasses for near work compared with boys at *p* < 0.05

### Lifestyle behaviour

Table 1 shows scores on time spent on near work, physical activity, and outdoor activities. Children in 10^th^ grade spent more time using digital devices (F(1,190) = 31.772, *p* < 0.001), doing schoolwork (on school tablet) (F(1,190) = 26.835, *p* < 0.001), and were less outdoor (F(1,190) = 10.219, *p* = 0.002), compared with children in 5^th^ grade. Further, children in 10^th^ grade read less on paper (F(1,190) = 5.650, *p* = 0.018), compared with the 5^th^ grade children. There were significantly fewer children in 10^th^ grade who walked or cycled to school (χ^2^(1, *n* = 192) = 9.49, *p* = 0.002) and were physically active during school breaks (χ^2^(1, *n* = 192) = 132.49, *p* < 0.001), compared with children in 5^th^ grade. In the total sample there were no significant correlations between time spent using digital devices and time spent on sports, physical activity, and outdoor activities. Girls had higher academic ambitions (F(1,139) = 11.255, *p* = 0.001), did more schoolwork (on school tablet) (F(1,190) = 5.846, p = 0.017) and read more on paper (F(1,190) = 6.662, *p* = 0.011), compared with boys. There were no significant differences between girls and boys regarding total time spent using digital devices, on sports and physical activity, or being outdoors.

Children with neck pain sometimes/often/always (*n* = 78) spent more time doing schoolwork on tablet (F(1,190) = 5.123, *p* = 0.025) and reading on mobile (F(1,190) = 6.022, *p* = 0.015) compared with the rest of the sample. Children with shoulder pain sometimes/often/always (*n* = 55) had significantly increased mobile use compared with the rest of the sample (F(1,190) = 4.694, *p* = 0.032). Children with low back pain often/always (*n* = 16), had increased total screen time (F(1,190) = 5.755, *p* = 0.017) and mobile use (F(1,190) = 6.622, *p* = 0.011). Further, children who reported headache during reading often/always (*n* = 12), spent significantly more time using digital devices (F(1,190) = 8.220, *p* = 0.005). There were no significant differences in socioeconomic status and total time spent on sports, physical activity, and outdoor activities, between children experiencing symptoms often/always or sometimes/often/always and the rest of the sample.

## Discussion

This study showed that laboured reading and upper body musculoskeletal pain were associated with non-compliance in wearing prescribed eye wear, increased use of near digital devices, and visual stress (glare).

The 15–16-year-old adolescents spent more time on digital devices and schoolwork, read less on paper, were less outdoor, and were less physically active during the school day (walking/cycling to school, physical activity during school breaks) compared with the 10–11-year-olds. This shift in behaviour from childhood to adolescence to a more indoor, sedentary, and screen-based lifestyle with higher academic demands, is in accordance with earlier studies [1, 4]. Concurrently, the frequency of reported laboured reading, eye symptoms during use of digital devices, and upper body musculoskeletal pain were increased, and scores were higher in girls. Increase in pain symptoms from childhood to early adulthood has been reported in several studies [5–8], in addition to higher prevalence of spinal pain and headache in girls [6, 7, 38, 69]. In this study, non-compliance with the use of corrective eye wear and more visual stress (reflections/glare) during screen use were significantly associated with laboured reading and upper body musculoskeletal pain. Children not wearing the prescribed corrective eye wear, often/always experienced eyestrain and headache during reading and use of digital devices, together with pain in shoulders and arm/wrist/hand. In line with this, studies have reported significant correlations between uncorrected vision and impaired academic performance in children and adolescents [48–58]. A study in 944 school-aged children showed an association between early literacy and visual acuity; children who adhered to spectacle wear improved their visual acuity and had the potential to improve literacy [55]. In a study with 1298 school children aged 8 years, hyperopia (far-sightedness) was associated with decreased reading and writing skills [49]. Uncorrected vision problems in children and adolescents have also been linked to increased frequency of both headache and neck, shoulder, and back pain [14, 17, 22, 23]. In a recent study, 10–15-year-old children presenting with headache and neck-, shoulder-, and back pain, had significantly more uncorrected vision problems and lacked necessary corrective eye wear for near, compared with a control group [17]. A study examining 10–13-year-old healthy children with corrected vision, showed that the children with the best visual acuity had less shoulder pain during screen use compared to those with poorer visual acuity [16]. Further, in accordance with this study, glare during screen work have been shown to affect neck- and shoulder musculature [70–72], and symptoms of photophobia/glare may be related to impaired binocular vision [73].

Girls reported more laboured reading and pain symptoms, higher academic ambitions, more time used on schoolwork and reading, in line with previous studies [74–77]. Girls were more likely to use eye wear for near work, compared with boys. Near work glasses are typically prescribed when a child has problems with sustained near work due to hyperopia and accommodative and binocular dysfunctions. A correct pair of near work glasses improve reading, writing and screen viewing and may prevent or relieve eye symptoms, headache and neck-, shoulder-, and back pain [78, 79]. Most Nordic school children are hyperopic, with no reported sex differences [19–21, 68, 78, 80]. The girls in this study were more occupied with intense and high-focused near work (schoolwork and reading), compared with boys, and this may have influenced the higher use of near work glasses among the girls. Further, uncorrected vision problems in combination with increased academic demands, may explain the increased prevalence of laboured reading and upper body musculoskeletal pain in the children who did not wear their recommended glasses/contact lenses (12 girls (11 in 10^th^ grade), 5 boys (4 in 10^th^ grade)) [77]. Increased frequency of pain symptoms in girls compared with boys, has previously been linked to differences in physiology and hormone profiles, and psychosocial factors [35, 81–83].

In the present study, headache and neck-, shoulder- and low back pain were also associated with increased use of near digital devices. Several studies in children and adolescents have showed correlations between duration of screen time and spinal pain [16, 34, 39, 84–86]. A study including 45 555 Danish children (2010–2014) showed more pronounced associations between spinal pain and screen time, compared to between spinal pain and physical inactivity [34]. There are few studies investigating pain symptoms in relation to the specific use of different types of digital devices in children and adolescents. However, children have been shown to hold their mobile close and only supported by their hands [16, 87, 88], and one study showed an inverse correlation between mobile viewing distance and neck pain [16]. Shorter viewing distances increase the load on the visual system and binocular vision, increasing the risk of eyestrain, headache and upper body musculoskeletal pain [25, 87, 89, 90].

In summary, this study supported the notion that near tasks, such as use of digital devices, are demanding for ocular muscles and the precise coordination between the visual system and the head-stabilizing muscles. Eye, musculoskeletal and headache symptoms may be aggravated with increased screen time, uncorrected vision problems and visual stress, such as glare [9–18, 70–72].

### Strengths and limitations

A strength of this study was that although the data were from a relatively small sample, the study population of 192 Norwegian children and adolescents was representative for 5^th^ and 10^th^ grade school children in Norway [64, 65], strengthening the potential generalizability of the study results to healthy Norwegians of the same age. Digital devices (tablet) were already implemented in teaching in Kongsberg municipality at the time of the data collection (2017). In 2022 the use of digital devices in Norwegian schools has further escalated, and many children use a tablet or a computer as their main tool for school- and homework. Combined with even easier access to private digital devices and increased use during the COVID-19 pandemic, this study probably underestimates screen time and the frequency of symptoms [91]. Study limitations included self-reported symptoms and time spent on different activities, which could bias the results. Also, we did not record systemic diseases, learning disabilities (e.g. dyslexia), developmental delays, or psychosocial variables, such as stress and quality of life, known to be associated with the experience of symptoms and pain in children and adolescents. Children with reading disabilities were likely to be equally present among the children with normal vision / adequate prescribed eye wear and children with non-compliance in wearing prescribed eye wear. Because of the cross-sectional design, the study was restricted to investigate contemporary associations in the data, and no conclusive statements regarding causal directions of the associations have been made. More research is required to understand the associations between types of refractive error, non-compliance, and development of symptoms.

## Conclusions

In this study, 15–16-year-olds reported to have a more indoor, sedentary, digital-based lifestyle with higher academic demands than 10–11-year-olds. Concurrently, experiences of laboured reading and upper body musculoskeletal pain increased with age, and girls were more affected than boys. Non-compliance in wearing prescribed eye wear, visual stress (glare), and increased use of digital devices aggravated these symptoms. Uncorrected vision problems challenge the ability to sustain long hours of static, intensive near work, and may provoke pain symptoms. This emphasizes the need for regular eye examinations in children and adolescents to ensure proper corrective eye wear if needed.

## Data Availability

The dataset analysed during the current study is available in the usn.figshare.com repository, https://doi.org/10.23642/usn.19045460
