# Correction: Laboured reading and musculoskeletal pain in school children - the role of lifestyle behaviour and eye wear: a cross-sectional study

**DOI:** 10.1186/s12887-022-03513-w

**Published:** 2022-07-28

**Authors:** Hanne-Mari Schiøtz Thorud, Randi Mork, Cecilie Onshuus Bjørset, Stuart J. Gilson, Lene A. Hagen, Trine Langaas, Hilde R. Pedersen, Ellen Svarverud, Gro Horgen Vikesdal, Rigmor C. Baraas

**Affiliations:** National Centre for Optics, Vision and Eye Care, Department of Optometry, Radiography and Lighting Design, University of South-Eastern Norway, Kongsberg, Norway

**Correction: BMC Pediatr 22, 416 (2022)**

**https://doi.org/10.1186/s12887-022-03465-1**

In the originally published version of this article [1] the word ‘undefined’ was incorrectly included in the second column in Table 2. The original article has been corrected.
